# Synthesis and characterization of chitosan-grafted-dopamine based micelles as multifunctional nanomedicines for Parkinson’s disease treatment by intranasal administration

**DOI:** 10.1007/s13346-025-01963-0

**Published:** 2025-09-01

**Authors:** Adriana Trapani, Annalucia Carbone, Sante Di Gioia, Giuseppe Fracchiolla, Piera Soccio, Filippo Maria Perna, Andrea Francesca Quivelli, Gian Paolo Suranna, Roberto Grisorio, Chiara Lo Porto, Daniele Conelli, Giuditta Colangelo, Massimo Conese

**Affiliations:** 1 Department of Pharmacy-Drug Sciences, University of Bari Aldo Moro, via E. Orabona, 4-70125 Bari, Italy; 2 Department of Clinical and Experimental Medicine, University of Foggia, 71122 Foggia, Italy; 3 Department of Medical and Surgical Sciences, University of Foggia, 71122 Foggia, Italy; 4 Department of Civil, Environmental, Land, Building Engineering and Chemistry (DICATECh), Bari Polytechnic, E. Orabona Street,4, I-70125 Bari, Italy

**Keywords:** Amphiphilic chitosan derivatives, *O*-Carboxymethyl-chitosan-*g*-dopamine conjugates, Self-assembly, Oxidative stress, Quantitative real time-PCR, Neuroinflammation

## Abstract

The main aim of this work was to synthesize new chitosan amphiphilic derivatives able to self-assembly encapsulating substances acting at different target sites implicated in Parkinson disease (PD). For this purpose, *O*-carboxymethyl-chitosan (*O*-CMCS) was grafted with dopamine (DA) exploiting a carbodiimide mediated coupling reaction using different polymer/DA weight ratios. The structural characterization of the resulting *O*-CMCS-*g*-DA conjugates was carried out by spectral (i.e., ì FT-IR, ^1^H-NMR spectroscopy) and potentiometric titrations. The physicochemical characterization of these conjugates was performed by thermal analysis, scanning electron microscopy coupled with energy dispersive X-ray spectroscopy. The in vitro DA release was carried out in simulated nasal fluid showing in any case a sustained release of the neurotransmitter. The amphiphilic *O*-CMCS-*g*-DA conjugate at highest substitution degree was allowed to form micelles using the dialysis method. The Critical Micellar Concentration of such micelles was determined by the conductometric method and resulted of 1 × 10^− 4^ mg/mL Quercetin (QUE), selected as hydrophobic antioxidant model drug, was encapsulated into the core of these micelles with an efficiency of 18%. From a biological point of view, none of the *O*-CMCS-*g*-DA conjugates was cytotoxic against the target neuronal SH-SY5Y cells. Moreover, all the *O*-CMCS-*g*-DA conjugates were able to modulate neuroinflammation as demonstrated by mRNA expression level analysis. Therefore, these *O*-CMCS-*g*-DA based micelles showed a great potential as multifunctional nanomedicines for brain delivery by intranasal route of a lipophilic antioxidant involved in the oxidative stress together with the neurotransmitter DA exploiting a delivery system with modulating properties of neuroinflammation.

**Supplementary Information:**

The online version contains supplementary material available at https://doi.org/10.1007/s13346-025-01963-0.

## Graphical abstract

**Figure:**
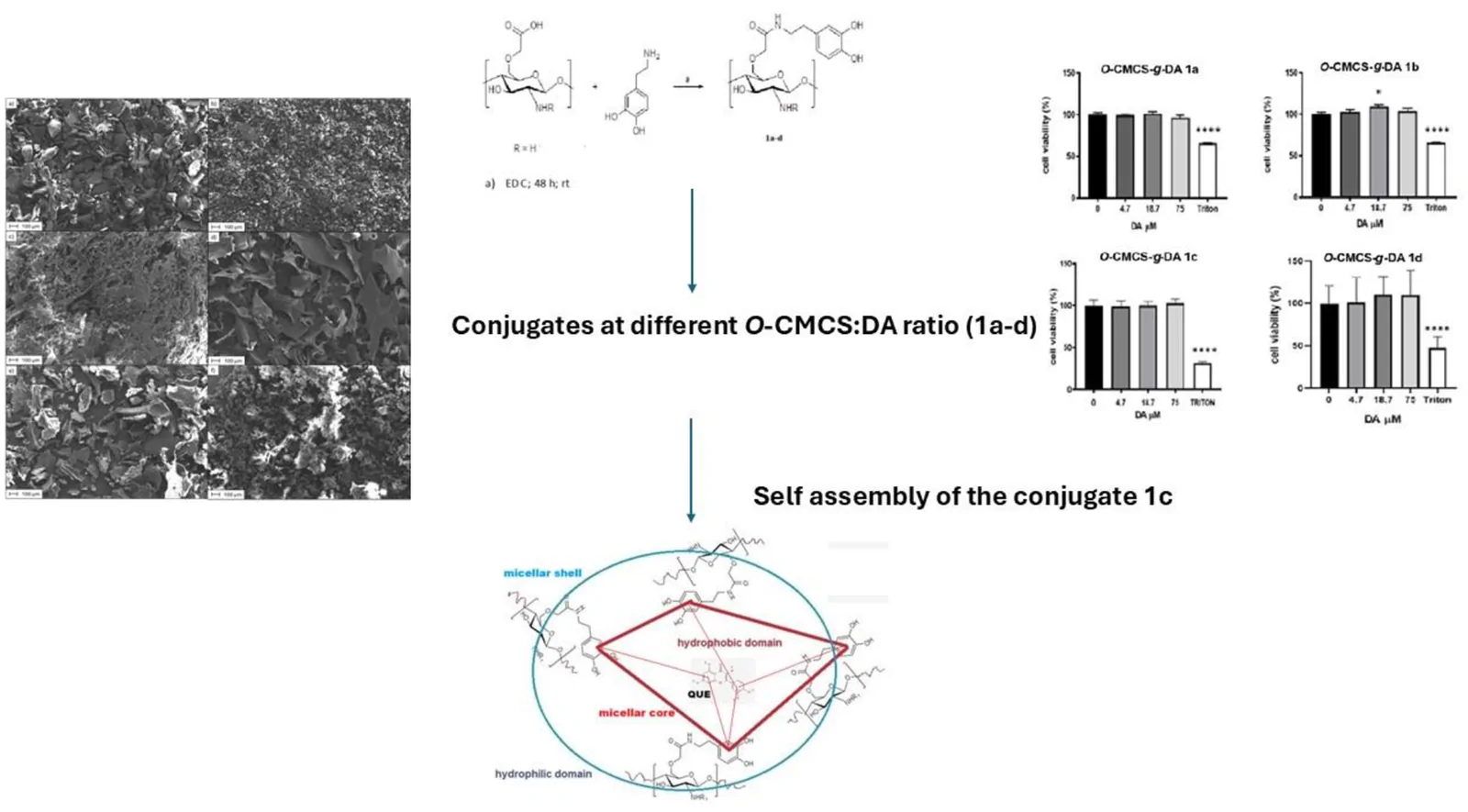

## Introduction

Among the numerous neurodegenerative diseases, including Alzheimer’s disease, Parkinson’s disease (PD), strokes, Huntington’s disease etc., PD showed a high incidence in the decades 1999–2019. According to the World Health Organization, indeed, in 2019 the global occurrence of PD has been calculated to be over 5.8 million cases [1] and moreover, the rate of PD is expected to double by 2030 due to the aging of the global population [2]. PD is a serious and the most common age-neurodegenerative disease after Alzheimer’s causing both motor and non-motor impairments. The former disabilities include tremors, bradykinesia, rigidity, and postural instability, while the latter ones concern cognitive decline, mood and sleep disorders. All these impairments, taken together, deeply affect the quality of life of the parkinsonian patient. It is recognized that PD is mainly related to the degeneration of dopamine (DA) neurons in the *Substantia nigra*, which plays a crucial role for movement control. Hence, the loss in dopamine levels in the in the nigrostriatal neurons constitutes the hallmark for motor symptoms of PD [3, 4]. Unfortunately, due to its unfavourable physicochemical properties, the neurotransmitter DA is unable to overcome the Blood Brain Barrier (BBB) and, therefore, the bio-precursor of DA, L-Dopa, is employed for the so called “dopamine-replacement therapy” because, after oral administration, indeed, it is able to bypass the BBB providing therapeutic levels of neurotransmitter in the brain of the PD patient [4]. Hence, dopamine replacement therapy is the most important treatment for PD and L-Dopa still constitutes the gold standard treatment of this neurodegenerative disease improving both the symptoms and life prognosis of patients. However, it was noted that the prolonged use of L-Dopa led to motor complications, such as a wearing-off phenomenon consisting in the re-appearance of motor and non-motor symptoms during L-Dopa free interval, providing a significant negative impact on the quality of life of PD patient [4]. To reduce the drawbacks of L-Dopa based therapy, dopamine agonists were developed leading to drugs which, as L-Dopa, are used for a symptomatic treatment of PD. However, in parallel to these studies, it became increasingly clear that, to effectively prevent dopamine neuron damage, an improvement of our understanding of the PD pathophysiology was necessary and, advancements in this direction, allowed the identification of new therapeutic targets useful for disease-modifying therapy and hinder the PD progression [4]. Thus, we now know that PD is a heterogeneous disease for which several risk factors are associated such as ageing, environmental factors, genetic and family history and that men are more susceptible than women [5, 6]. Further therapeutic targets identified for PD disease-modifying therapy include dysfunction in mitochondria or lysosomes, formation of toxic aggregates of α-synuclein (α-syn), neuroinflammation, oxidative stress, and other issues [5, 7, 8]. In particular, it is assumed that chronic neuroinflammation plays a pivotal role in initiating and/or or accelerating the progress in the detrimental cascade that leads to DAergic cell loss. Inflammatory mediators, such as cytokines/chemokines, are produced by different cell types (*e*,* g*. neurons, microglia, T cells), following cell autonomous alterations in neuronal cells, including mitochondrial damage and protein aggregation, and thus aggravating neurodegeneration [9].

Despite these targets represents a significant advancement in the PD treatment, the effective delivery of therapeutic agents to hit them still represents a significant challenge for researchers involved in the field [3, 5]. Indeed, in the brain there are physiological barriers, mainly the BBB, which rule out potential therapeutic agents from entering the brain following systemic administration, hindering so the attainment of their target in the CNS. Hence, the inability of several potentially active substances at brain level to overcome obstacles as the BBB in therapeutic amounts mainly explains their failure in treating brain disorders.

To avoid this problem, in recent years, most attention has been focused on the administration of drugs by intranasal route that constitutes a non-invasive method to by passes the BBB providing direct access of drugs to the CNS using principally the anatomical connections between the nasal respiratory region and the olfactory bulb or the trigeminal nerve [5, 10, 11]. Compared with the oral administration route, the intranasal administration can provide faster brain delivery, avoid gastrointestinal and hepatic metabolism and reduce systemic side effects. However, following the nose-to-brain (NTB) pathway, some limitations can occur as, for instance, the presence of mucociliary clearance, which reduce the residence time with nasal mucosa, the poor permeation of hydrophilic drugs and the enzymatic degradation in the nasal cavity [5, 10, 11].

An interesting outcome, arising from wide investigation in the field, was the established ability of appropriate nanosized drug delivery systems to overcome the limitations presented by NTB [5, 10]. Thus, the encapsulation in nanocarriers can allow not only the protection of the nano-encapsulated drug from the enzymatic degradation in the nasal cavity but also to facilitate the BBB crossing and targeted drug accumulation within the brain. Various polymeric and inorganic nanoparticles have shown noteworthy efficiency in the PD treatment [5, 10]. Similarly, lipid based nanocarriers such as liposomes, nanogels, micelles, solid lipid nanoparticles and nanostructured lipid carriers have shown sustained release of therapeutic agents, enhanced bioavailability, and reduced side effects. Ultimately, it can be stated that to avoid or cross the BBB the choice of the appropriate drug administration route and delivery method plays a key role.

The significant advancement made in biomaterials utilized for the preparation of nanocarriers led to systems more compatible vehicles with biological systems, enhanced therapeutic efficacy and smaller toxicity. Moreover, the progress in the biomaterial field also enabled the utilization of nanomaterial formulations able to reduce oxidative stress and neuroinflammation offering promising new options for diagnosis and therapeutic treatment of PD [3, 5, 6]. In this regard, it is useful to mention the innovative approach recently introduced for the treatment of brain diseases and denoted as “multifunctional nanomedicines”. It consists in using nanocarriers able to involve multiple disease fronts by the co-delivery of two or more active substances acting at different target sites [12]. In other words, such multifunctional nanomedicines are able to accomplish multiple biological roles by the co-delivery of multiple cargos with different therapeutic and/or diagnostic properties combined into a single nanocarrier to the brain parenchyma [12].

For brain delivery, several biopolymers, i.e., polymers obtained from animal, plant, or microbial sources, have been employed to prepare vehicles that carry drugs or diagnostic agents, cross the BBB and reach therapeutic target sites since they offer several advantages including low toxicity, biodegradability, biocompatibility and mucoadhesive properties [13]. Among the biopolymers currently used, chitosan (CS) has demonstrated significant potential for delivering drugs to the brain not only because it is endowed with low toxicity, biodegradability, biocompatibility and mucoadhesive properties but also because it can be easily functionalized leading to CS-based biopolymers to be used as vehicles for brain delivery. CS, indeed, has many functional groups, as -NH_2_ and primary and secondary -OH groups, enabling a wide range of chemical modifications such as acylation, tosylation, and *O*-carboxymethylation [14, 15]. Besides this, the advantages of using CS-based nanoparticles by intranasal route as a promising way for management of neurodegenerative disorders, including PD, are also documented in literature [16].

In this context, we recently reported the synthesis of the amphiphilic polymers *N*,* O*-carboxymethylchitosan-grafted-Dopamine (*N*,*O*-CMCS-*g*-DA) and *O*-carboxymethylchitosan-grafted-Dopamine (*O*-CMCS-*g*-DA) by conjugation of the primary amino group of neurotransmitter DA with the –COOH function of *N*,*O*-CMCS and *O*-CMCS, respectively, through a carbodiimide mediated reaction in aqueous medium. These CMCS-*g*-DA conjugates were envisaged to self-assemble into polymeric micelles by evaporation method, nevertheless they provided polymeric nanoparticles in aqueous environment in most cases. As for the self-assembly process of *N*,* O*- or *O*-CMCS-DA conjugates, both of them may be considered amphiphilic polymers since hydrophobic domains can be envisaged due to specific π–π- as well as hydrogen bonding-interactions between the catechol moieties of these conjugates. Moreover, the better performance of *O*-CMCS-*g*-DA to provide multifunctional nanomedicines useful for intranasal anti Parkinson therapy was also evidenced [17]. However, even though polymeric micelles were expected in aqueous medium using *O*-CMCS-*g*-DA amphiphilic polymer as a result of π-π hydrophobic interactions between the catechol moieties just as happens for other phenolic compounds conjugated with *O*-CMCS [18], in the previous work we were unable to demonstrate such self-assembled system [17]. It was probably due to the low substitution degree (DS) of *O*-CMCS-*g*-DA conjugate previously prepared (i.e., 12.65%). Therefore, as a continuation of previous studies, the main aim of the present work was to prepare *O*-CMCS-*g*-DA conjugates at different polymer/DA (w/w) ratios with a DS sufficient to give self-assembly and provide stable polymeric micelles enabling the encapsulation of hydrophobic therapeutic agents in their core. For this purpose, we prepared *O*-CMCS-*g*-DA conjugates increasing DA amounts adopted in the synthetic procedure and the conjugate at highest DS was characterized by spectroscopic means, thermal analysis and in vitro DA release kinetic. Moreover, micelles were produced by dialysis method, and the critical micelle concentration was measured. To consider the translational capability of *O*-CMCS-*g*-DA conjugates, their bio/cytocompatibility and anti-inflammatory properties were evaluated in neuroblastoma SHSY-5Y cells.

## Materials and methods

### Chemical materials

*O*-Carboxymethyl Chitosan (*O*-CMCS, Molecular weight in the range of 100–300 kDa, deacetylation degree, 90%; substitution degree 88%, according to the technical sheet) was purchased from DBA (Milan, Italy). D_2_O, Ethyl-dimethyl-aminopropylcarbodiimide hydrochloride (EDC), dopamine hydrochloride (DA), Quercetin (QUE, free base), DMSO and phosphate buffer saline (PBS) were purchased from Sigma-Aldrich (Milan, Italy). Dialysis tubes with a MWCO 12–14 kDa and 3.5–4.5 kDa were purchased from Spectra Labs (Milan, Italy). Throughout this work, double distilled water was used. All other chemicals used were of reagent grade. Methanol for high-performance liquid chromatography (HPLC) grade was acquired from Levanchimica (Bari, Italy).

### Biological materials, cell line and culture

SHSY5Y cells (kindly provided by Dr. Maria Lasalvia, University of Foggia) were grown in Dulbecco’s Modified Eagle’s Medium Nutrient Mixture F-12 Ham (Sigma-Aldrich, Cat# D8062), 20% FBS, 1% non-essential amino acids, 2 mM L-glutamine, 1% penicillin and streptomycin, and 25 µg/mL amphotericin B. Cells were maintained at 37 °C and 5% CO_2_ in a humidified incubator and the culture medium was refreshed every other day.

### Methods

#### Apparatus

Fourier transform infrared (FT-IR) spectra of the conjugates prepared were obtained in KBr discs using a Perkin Elmer 1600 FT-IR spectrometer (Perkin Elmer, Milan, Italy). The range examined was 4,000–400 cm^− 1^ with a resolution of 1 cm^− 1^. Proton nuclear magnetic resonance (^1^H NMR) spectra of the conjugates prepared were recorded on a Bruker spectrometer (Billerica, Massachusetts, US) operating at 600 MHz and the analyses were performed at 293 K on dilute solutions of each conjugate using D_2_O as solvent. In particular, to allow the determination of the substitution degree of *O*-CMCS-*g*-DA *via*^1^H-NMR spectrometry, dispersions in D_2_O were prepared for conjugates at a concentration of 5 mg/mL adding 0.05 mL of HCl 10% in water (v/v) before the analysis. Chemical shifts are reported in parts per million (δ). Spectra were processed using Mnova (Mestrelab Research, A Coruna, Spain). For UV analysis, a Schimadzu 1900i UV-VIS spectrophotometer (Shimadzu, Milan, Italy) was used. Differential Scanning Calorimetry (DSC) runs were conducted using a Q2000 TA instrument (New Castle, DE, US) equipped with a TA Software.

Thermogravimetric analysis (TGA) was carried out on a Perkin-Elmer Pyris 6 TGA instrument in the range 30–600 ◦C under inert nitrogen flow (40 mL/min) at the heating rate of 20 ◦C/min.

HPLC analysis of DA was carried out with an instrument constituted by a Waters Model 600 pump (Waters Corp., Milford, MA), a Waters 2996 photodiode array detector and a 20 µL loop injection autosampler (Waters 717 plus).

The hydrodynamic mean diameters, polydispersity indexes and zeta potential values of *O*-CMCS-*g*-DA based polymeric micelles were determined by using a Zetasizer NanoZS, ZEN 3600 (Malvern Instruments, Worcestershire, U.K.) instrument according to photon correlation spectroscopy (PCS) mode.

#### Synthesis of *O*-carboxymethylchitosan-grafted-dopamine 1a-d

**Scheme 1:**
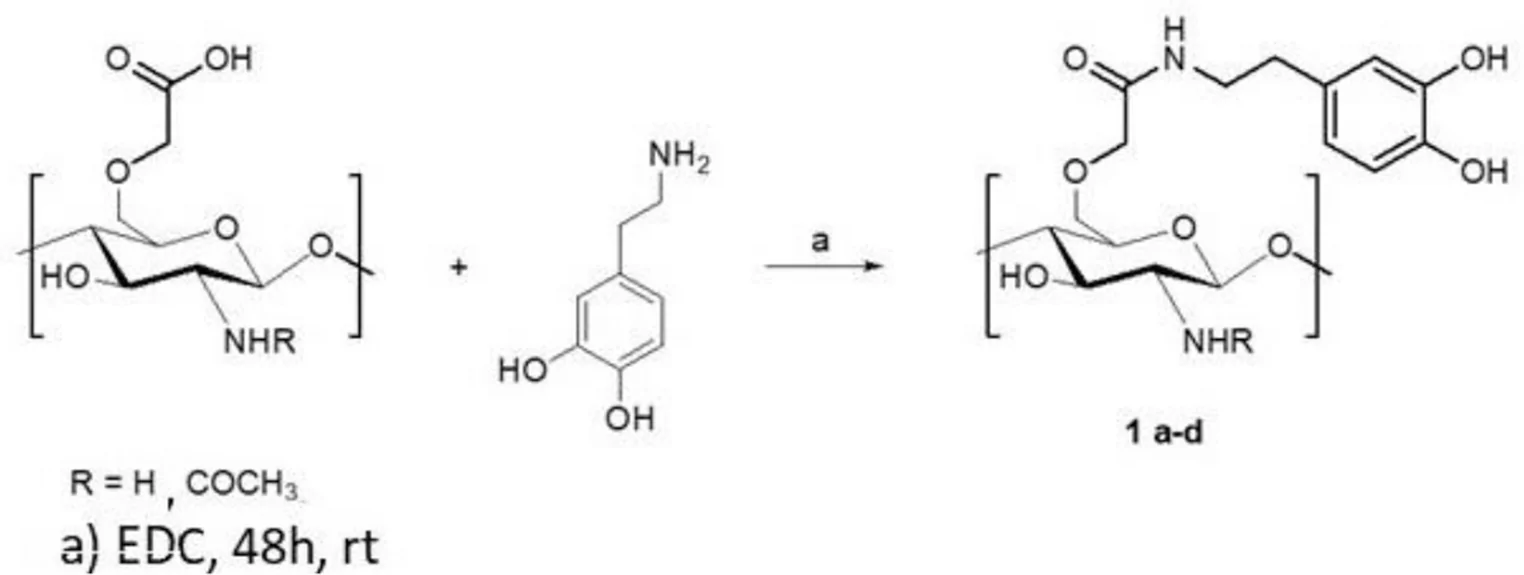
Synthetic pathway followed to obtain *O*-CMCS-*g*-DA amide conjugates **1a-d** employing different *O*-CMCS: DA (w: w) ratios

The synthesis of *O*-CMCS-*g*-DA conjugates **1a-d** was carried out through a carbodiimide mediated reaction in aqueous environment as showed in Scheme 1 and using the protocol described in the previously work [17]. Briefly, as for the preparation of conjugate **1a**, to a solution at pH 6 constituted of diluted HCl (0.1 N, 50 mL), 200 mg of *O-*CMCS were added, afterwords DA (200 mg) and EDC (50 mg) were also put in the reaction mixture. This last was left under magnetic stirring and under argon atmosphere for 48 h at room temperature (r.t.). Afterwards, the product was dialyzed in distilled water for three days and then lyophilized (Lio Pascal 5P, Milan Italy) and the obtained product (i.e.,* O-*CMCS-*g*-DA conjugate **1a**) was collected. Conjugates **1b-d** were similarly prepared. Conjugates **1a-c** after lyophilization were obtained as powders, while conjugate **1d** was obtained as sponge-like solid.

#### Characterization of *O*-carboxymethylchitosan-grafted-dopamine conjugates 1a-d

The characterization of *O*-CMCS-*g*-DA amphiphilic polymers was performed by FT-IR, ^1^H-NMR, degree of substitution (DS), TGA, DSC, SEM microscopy and, for the conjugate at highest DS **1c** only, by critical micelle concentration (CMC) evaluation.

#### Determination of substitution degree (DS) of each *O*-CMCS-g-DA conjugates 1a-d by ^1^H-NMR spectroscopy, or by hydrolysis under strong acidic or basic conditions

The percentage of monomeric units substituted with DA (DA_subst_) (i.e. DS value), was determined by ^1^H-NMR spectroscopy comparing the integral of the signals in the aromatic region (6.55–7.25 ppm) with that of the multiplets in the region 3.22–4.50 ppm. The latter region accounts for the H3, H4, H5, and 2 × H6 protons for both *O*-CMCS bonded with DA and free *O*-CMCS. The calculation used the following Eq. (1):

1 $${\rm{D}}{{\rm{A}}_{{\rm{subst}}}}{\rm{ = }}{{{\rm{Integra}}{{\rm{l}}_{{\rm{H - Ar}}}}{\rm{ \times 5}}} \over {{\rm{Integra}}{{\rm{l}}_{{\rm{H3,}}\,{\rm{H4,}}\,{\rm{H5,}}\,{\rm{H6}}}}}}$$

where Integral_H−Ar_ is the sum of the integrals for the signals in the region 6.55–7.25 ppm divided by three, and Integral_H3,H4,H5,H6_ is the intensity of the signals for H3, H4, H5, and both H6 protons. All the determinations of DS by ^1^H-NMR spectroscopy were carried out on each **1a-d** conjugates in triplicate and the obtained results are reported in as mean ± standard deviation (SD).

According to the hydrolysis under strong acidic conditions previously described [19], DS% in DA of each *O*-CMCS-*g*-DA conjugate **1a-d** was also measured. Briefly, 2 mg of each O-CMCS-*g*-DA conjugate were weighed and dissolved in HCl 1 N (pH 1) under stirring and light protection at r.t. for 3 h. Then, the solution was centrifuged (16,000× g, for 45 min, Eppendorf 5415D, Germany) and the obtained supernatant was analysed by HPLC to obtain the DA amounts. The DS% was calculated as mg DA/mg *O*-CMCS-g-DA conjugate x 100 [19]. All the determinations of DS by hydrolysis under strong acidic conditions were carried out on each **1a-d** conjugates in triplicate and the obtained results are reported in as mean ± SD.

In the case of *O*-CMCS-*g*-DA conjugate **1c**, the substitution degree (DS) was also determined by potentiometric titration after alkaline hydrolysis according to a procedure previously described with slight modification [20, 21]. *O*-CMCS-*g*-DA conjugate **1c** (50 mg) was dissolved in 5 mL of a solution 0.25 N of NaOH in ethanol and the mixture was stirred at reflux for 24 h. Then, at r. t. an acid-base potentiometric titration on the dark-yellow mixture (1 mL) was performed by the use of the automatic titrator (TITREX-1000, Wertheim, Germany) with a pH meter employing a standardized solution of HCl (0.1 N). The potentiometric titration was carried out in triplicate.

During titration, two equivalent points were determined. The first point referred headline in excess of NaOH (V1 equivalent point); the second equivalent point referred to the neutralization of the acid salt present (V2 equivalent point). The moles of HCl occurring between the first and the second equivalent point corresponding to the moles of free amide and the DS% were, therefore, determined by the following Eq. (2):

2 $${\rm{DS\% = }}\left[ {{\rm{mass }}\left({{\rm{sample}}} \right){\rm{ -- mass }}\left({{\rm{free\:amide}}} \right)} \right]{\rm{ \times 100/mass }}\left({{\rm{sample}}} \right)$$

where mass (sample) corresponds to 1 mL (10 mg) of each conjugate sample before alkaline hydrolysis; moles of free amide are equal to: (V2 equivalent point - V1 equivalent point) x [HCl] determined by potentiometric titration as the average of three titrations.

### Thermal analysis

Thermal analysis on the herein examined conjugates encompassed both studies of TGA and DSC.

Thermogravimetric analysis (TGA) was carried out on a Perkin-Elmer Pyris6 TGA instrument in the range 30–600 ^◦^C under inert nitrogen flow (40 mL/min) at the heating rate of 20 ^◦^C/min. For each sample, after weighting 5–10 mg, thermograms (TG) and corresponding derivative curves (DTG) were elaborated with the TGA Pyris software (version 8.0.0.0172 Waltham, USA) before plotting.

Differential Scanning Calorimetry (DSC) runs were conducted using a Q2000 TA instrument (New Castle, DE, US) equipped with a TA Software. Aliquots of about 5 mg of each analyte (i.e., pure DA, pure *O*-CMCS, *O*-CMCS-*g*-DA conjugates at different w/w ratios) were placed in an aluminum pan and hermetically sealed. The scanning rate was set at 5 °C/min under a nitrogen flow of 50 cm^3^/min and the temperature range was from 25 to 320 °C. The calorimetric system was calibrated in transition temperature by using indium (99.9% purity) and following the procedure of the TA Software. Each DSC thermogram was acquired in triplicate to check the reproducibility.

### Morphology of *O*-CMCS-g-DA conjugates 1a-d by SEM analysis

The morphology of the **1a-d** conjugates and controls DA and *O*-CMCS was analysed by scanning electron microscopy (SEM) coupled with energy dispersive X-ray spectroscopy (EDX) using a Zeiss Σigma 300VP electron microscope equipped with an Oxford C-MaxN SDD detector with an active area of 20 mm^2^. Prior to the analysis, a metallization with gold was carried out on all samples. To enhance conductivity and prevent charging artifacts during SEM imaging, gold for sputter coating was selected, due to its excellent electrical conductivity and chemical inertness, which also preserves the sample morphological details. In details, all tested **1a-d** conjugates and controls DA and *O*-CMCS were subjected to gold sputtering layer using a Quorum Q150R ES sputter coater. The process was conducted in a high-purity (99.998%) nitrogen atmosphere.

### In vitro DA release from *O*-CMCS-g-DA conjugates 1a-d in simulated nasal fluid (SNF)

In view of an intranasal administration, the in vitro DA release in SNF [22] (pH 6.0, without enzymes) from all tested conjugates was carried out starting from a weighted amount of each conjugate corresponding to 1-1.2 mg of DA and dispersing it in 2.5 mL of SNF containing 0.05% (v/v) of DMSO. The assay was performed at 37 ± 0.1 °C in an agitated (40 rpm/min) water bath (Julabo, Milan, Italy) for 14 days. At scheduled time points, 0.25 mL of the receiving medium were withdrawn and replaced with 0.25 mL of fresh medium. Then, each sample was centrifuged at 16,000 × g for 45 min, (Eppendorf 5415D, Germany), and the amounts of the delivered DA were quantified in the resulting supernatants by HPLC, as below described, and plotted against the time. All the release experiments in SNF were carried out in triplicate for each conjugate.

The DA quantitative determination was performed by HPLC apparatus above mentioned and employing the previously reported conditions [23, 24]. Briefly, the mobile phase was formed by 0.02 M potassium phosphate buffer, pH 2.8: CH_3_OH 70:30 (v/v), and the elution of the column in isocratic mode occurred at the flow rate of 0.7 mL/min. Data were processed by the Empower™ Software Build. For analysis, the stationary phase was a Synergy Hydro-RP (25 cm 4.6 mm, 4 μm particles; Phenomenex, Torrance, CA) column in conjunction with a precolumn C18 insert. Standard calibration curves for DA determination were obtained at 280 nm wavelength dissolving DA in the eluent above mentioned. Under such chromatographic conditions, DA retention time was of 5.5 min, whereas the retention time of the examined conjugates was of 4.9 min.

For *O*-CMCS-*g*-DA conjugate **1c**, the in vitro DA release was also carried out in PBS (pH 7.2) over 7 days upon the same conditions above described for the assay in SNF.

### Preparation of unloaded *O*-CMCS-g-DA micelles from *O*-CMCS‐g-DA conjugate 1c

Unloaded polymeric micelles starting from *O*-CMCS-*g*-DA conjugate **1c** were prepared according to the dialysis method [25–27]. Briefly, 20 mg of the *O*-CMCS-*g*-DA conjugate **1c** were dispersed in 20 mL of SNF under stirring at r.t. for 24 h. Then, the mixture was introduced in a dialytic bag (MWCO 3.5–4.5 KDa) and let to dialyze against double distilled water over 48 h. Once finished, the mixture in the dialysis bag was centrifuged at 13,200 rpm for 45 min (Eppendorf 5415D, Germany) and the pellet was discarded, the corresponding supernatant was isolated to measure particle size and zeta potential values by using the ZetasizerNanZS (ZEN 3600, Malvern, UK) apparatus. Particle size and polydispersion index (PDI) values of the unloaded *O*-CMCS-*g*-DA micelles were measured at 25 °C by redispersing each of them in 1 mL of double distilled water followed by a brief sonication. Then, the samples underwent to a further dilution in double distilled water (1:20, v/v) prior to be analyzed for particle size. For zeta-potential measurements laser Doppler anemometry technique was adopted (ZetasizerNanoZS, ZEN 3600, Malvern, UK) by using the same dilution as above described for size analysis.

### Determination of the critical micelle concentration (CMC) for the *O*-CMCS-g-DA conjugate 1c

The CMC of the *O*-CMCS-*g*-DA conjugate **1c** was determined by conductimetric titration (CON110/Handheld Conductivity/TDS/Temperature/RS232C Meter; Eutech Instruments, Thermo Fisher Scientific, Milan, Italy) performed at 25 °C [28, 29]. The procedure is based on the conductivity measurement of various conjugate concentrations in double distilled water in the range between 1 × 10^− 2^ and 1 × 10^− 7^ mg/mL. The CMC corresponds to the concentration at which there is an abrupt change of slope of the curve plotted by the specific conductivity against the logarithm of the concentration of the conjugate.

### Preparation of quercetin-loaded *O*-CMCS-g-DA micelles from *O*-CMCS‐g-DA conjugate 1c

As above, 20 mg of the *O*-CMCS-*g*-DA conjugate **1c** were dispersed in 20 mL of SNF under stirring at r.t. for 24 h. Once the *O*-CMCS*-g*-DA conjugate **1c** mixture filled, as above, the dialysis bag, it was let to dialyze over 48 h *versus* a medium composed of ethanol: water 1:1 (v: v) containing QUE at the concentration of 1 mg/mL. Afterwords, once dialysis was finished, the mixture in the dialysis bag was centrifuged, as above, in order to isolate QUE-loaded *O*-CMCS-*g*-DA conjugate **1c** based micelles. Particle size and polydispersion index (PDI) values of QUE-loaded *O*-CMCS-*g*-DA micelles were measured at 25 °C by diluting them in double distilled water (1:20, v/v) prior to be analyzed for particle size by PCS.

### Quantitative determination of quercetin

The quantitative determination of QUE was carried out by spectrophotometric analysis using a calibration curve obtained by dissolving QUE in ethanol (concentration range 11–0.11 µg/mL, R^2^ > 0.999). The measurements were performed at the wavelength of 375 nm. The encapsulation efficiency of QUE in the conjugate *O*-CMCS-DA **1c** based micelles (E.E.%) was calculated by Eq. (3):

3 $$\eqalign{& {\rm{E}}{\rm{.E}}{\rm{.\% }} \cr & {\rm{ = }}\,{\rm{QUE}}\,{\rm{in}}\,{\rm{the}}\,{\rm{supernatant}}\,{\rm{after}}\,{\rm{centrifugation/Total}}\,{\rm{QUE*100}} \cr} $$

where total QUE is intended as the starting amount of the antioxidant. The study was performed in triplicate.

### Biocompatibility evaluation of *O*-CMCS-g-DA conjugates 1a-d with SH-SY5Y cell model line

SH-SY5Y cells were grown as previously described [30]. Briefly, cells were plated in a 96-well plate (BD, USA) at a density of 1 × 10^4^ per well in 100 µL of complete medium and incubated overnight to allow cell attachment. Then, the culture medium was replaced with 100 µL of fresh medium containing different dilutions of *O*-CMCS-*g*-DA conjugates **1a-d** (final concentrations of DA: 75 µM, 18.75 µM and 4 µM). In each experiment, untreated cells were used negative controls, whereas 1% Triton-X100-treated cells were used as a positive control. Under these conditions cells were grown at 37 °C for 24 h. After treatments, cell viability was evaluated by MTT (3-(4,5-dimethylthiazol-2-yl)-2,5 diphenyl tetrazolium bromide), as previously described [22]. The cell viability was calculated as follows:

%viability = [(Optical density {OD} of treated cell – OD of blank)/(OD of vehicle control – OD of blank)×100 ], considering untreated cells as 100%.

### Cytokines detection by quantitative real time-PCR (qRT-PCR)

SH-SY5Y cells were plated in a 6-well plate (Vazyme, Red Maple Hi-tech Industry Park, Nanjing, PRC, Cat# CCP01006) at a density of 5 × 10^5^ per well in 2 mL of complete medium and incubated overnight to allow cell attachment. Then, the culture medium was replaced with 2 mL of fresh medium containing different *O*-CMCS-*g*-DA conjugates **1a**, **1b**, **1c** and **1d** at the final concentration of dopamine 75 µM, corresponding to 11.5 µg/mL. Under these conditions cells were grown at 37 °C for 24 h. After treatments, RNA extraction was performed using the standard TRIzol reagent method (Thermo Fisher Scientific, Waltham, MA, USA, Cat# 15596018), following the manufacturer’s recommended protocol. Subsequently, RNA concentration and quality were evaluated using the NanoDrop 1000 spectrophotometer (Thermo Fisher Scientific, Italy). The absorbance ratio at OD260/OD280 was employed to assess RNA purity. Reverse transcription was performed using a High-Capacity cDNA Reverse Transcription Kit (Thermo Fisher Scientific, Cat# 4366596) according to the manufacturer’s instructions. TaqMan-based real-time PCR reactions were performed using AceQ Universal U + Probe Master MiX V2 (Vazyme, Cat# Q513-02) and TaqMan probes (ThermoFisher Scientific, Cat# 4331182, Italy) according to the manufacturer’s instructions, with the following program: (i) 37 °C for 10 min; (ii) 95 °C for 5 minutes; (iii) 95 °C for 10 s and 60 °C for 30 s, repeated for 45 cycles. Real-time PCR reactions were carried out in duplicate for each sample using 96-well plates and the reactions were performed on the ABI-PRISM 7300 instrument (PE Applied Biosystems, Waltham).

Relative quantification was conducted using the comparative 2 − ΔΔCt method, with β-actin serving as a normalizer. A list of genes with the relative primers code is reported in Table 1.

**Table 1:** Probes used for qRT-PCR

Gene symbol	Assay ID
Actb	Hs99999903_m1
IL1b	Hs01555410_m1
Tnf	Hs00174128_m1
Il6	Hs00174131_m1
Cxcl8	Hs00174103_m1

### Statistical analysis

Statistical analyses were carried out by Prism v. 5, GraphPad Software Inc., USA. Results were expressed as mean ± SD. Multiple comparisons were based on one-way analysis of variance (ANOVA) with Bonferroni or Tukey post hoc test and differences were considered significant when *p* < 0.05.

## Results

### Synthesis and characterization of *O*-carboxymethylchitosan-grafted-dopamine conjugates 1a-d

*O*-CMCS-*g*-DA conjugates **1a-d** were prepared by an EDC mediated amidation reaction occurring between the -NH_2_ group of the neurotransmitter DA and the–COOH group of the starting polymer *O*-CMCS in aqueous environment (Scheme 1). In particular, the reagents (i.e., polymer and DA) as well as the coupling agent EDC were employed in the weight ratio showed in Table 2 and the reaction mixture was kept under stirring at r.t. for 48 h according to that previously described [17]. Then, the obtained conjugates were purified by dialysis in distilled water for three days and lyophilized. Thus, it was possible to graft DA moieties on the *O*-CMCS backbone at an acidic pH at which the neurotransmitter is not prone to autoxidation reaction [31]. The structural assignment of the obtained conjugates was confirmed by FT-IR and ^1^H NMR.

**Fig. 1:**
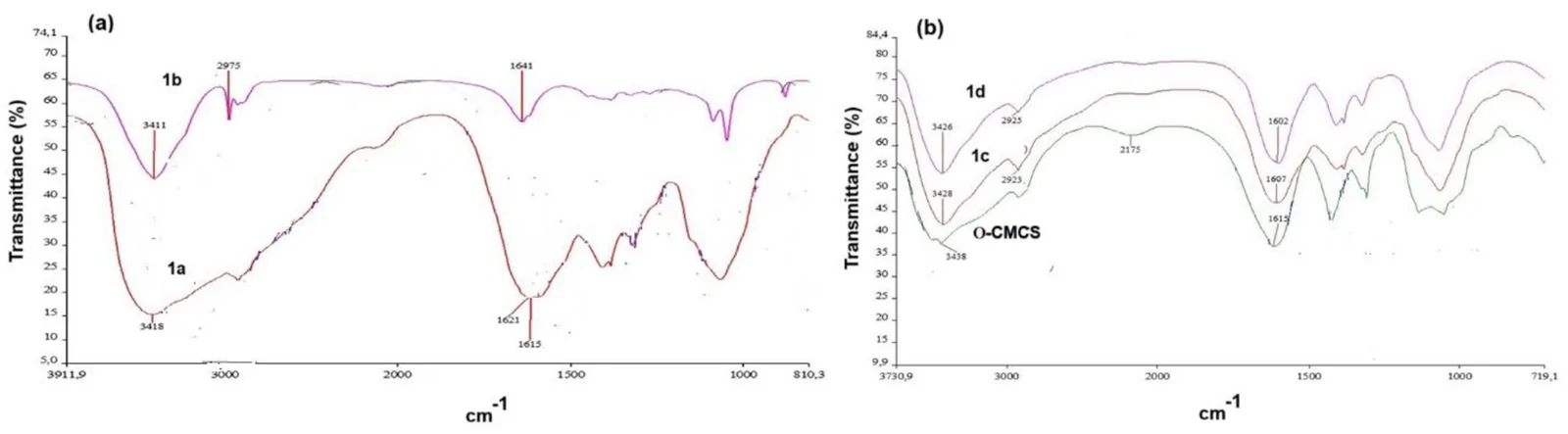
(**a**) FT-IR spectra of *O*-CMCS-*g*-DA conjugates **1a** and **1b**; (**b**) FT-IR spectra of *O*-CMCS-*g*-DA conjugates **1c**, **1b** and pure *O*-CMCS

In the FT-IR spectra of the *O*-CMCS-*g*-DA conjugates **1a-d**, the absorption bands attributable to the amide carbonyl groups can be identified in Fig. 1a, b in the range 1602–1641 cm^− 1^. In addition, the characteristic bands that were assigned to O-H superimposed with N-H stretching vibrations occurred in the range 3411–3428 cm^− 1^, while those attributable to the C-H aliphatic absorption bands were also identified in the range 2923–2975 cm^− 1^.

**Table 2:** Polymer/DA weight ratio used in the synthesis, yields and substitution degree (DS), of *O*-CMCS-*g*-DA conjugates **1a-d**

O-CMCS-g-DA conjugate	polymer/DA ratio (w/w)	EDC (mg)	Yield (%)	DS ^a^ (%, by ^1^H-NMR)	DS (%, by acidic hydrolysis) ^b^	DS (%, by basic hydrolysis) ^c^
**1a**	1:1 (1) (50 mg/50 mg)	12.5	26 (± 1)	14 (± 2)	16 (± 2)	
**1b**	1:2 (0.5) (50 mg/100 mg)	12.5	20 (± 5)	24 (± 2)	36 (± 8)	
**1c**	1:3 (0.33) (33 mg/100 mg)	8	17 (± 2)	27 (± 2)	40 (± 4)	20.3 (± 0.3)
**1d**	2:1 (2) (50 mg/25 mg)	12.5	38 (± 8)	11 (± 2)	18 (± 3)	
**1e** ^d^	(0.714) ^d^ (150 mg/210 mg) ^d^	38 ^d^	24 (± 2) ^d^	12.65 ^d^	6 (± 0.9) ^d^	

^a^ DS obtained by ^1^H-NMR spectroscopy; ^b^ DS obtained by hydrolysis under strong acidic conditions; ^c^ DS obtained by hydrolysis under strong basic conditions. ^d^ From Ref [17]

Noteworthy, in all the FT-IR spectra of *O*-CMCS-*g*-DA conjugates **1a-d**, no absorption band at 1730 cm^− 1^ occurred suggesting that in these chitosan derivatives the carboxymethyl substituent is present in – COONa form [20]. The FTIR spectra of *O*-CMCS-*g*-DA conjugates **1a-d** show overall similarity to the parent *O*-CMCS spectrum, whereas absorption bands linked to the presence of the catechol moieties were no detected (Fig. 1a, b).

**Fig. 2:**
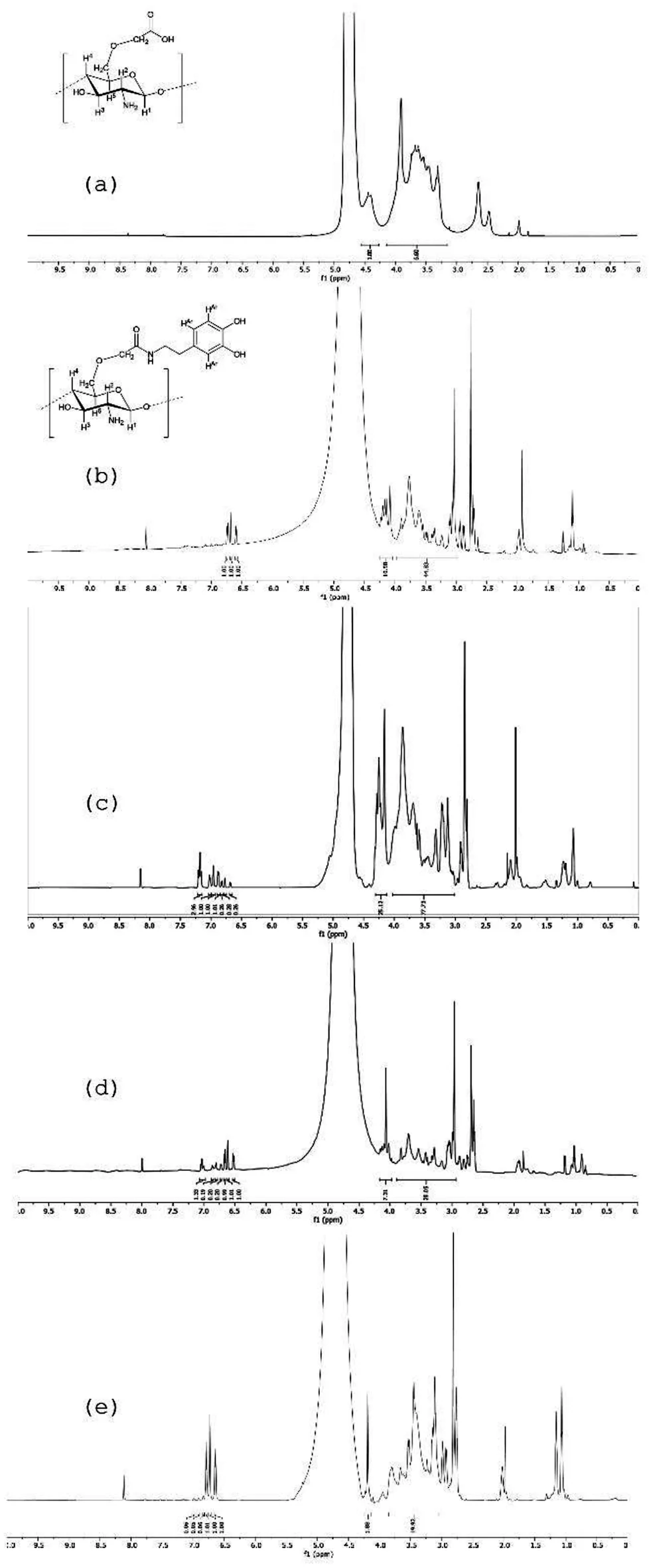
^1^H NMR spectrum of **a**) ^1^H-NMR spectrum of the polysaccharide *O*-CMCS in D_2_O, (**b**) ^1^H-NMR spectrum of *O*-CMCS-*g*-DA conjugate **1d**, (**c**) ^1^H-NMR spectrum of *O*-CMCS-*g*-DA conjugate **1a**, (**d**) ^1^H-NMR spectrum of *O*-CMCS-*g*-DA conjugate **1b**, (**e**) ^1^H-NMR spectrum of *O*-CMC-*g*-DA conjugate **1c**

^1^H NMR also confirmed the grafting of DA moieties on the *O*-CMCS backbone. From the analysis of Fig. 2a, showing the ^1^H NMR spectrum of the polysaccharide *O*-CMCS in D_2_O, multiplets from 3.91 to 3.27 ppm were found that were assigned to protons H3, H4, H5, H6 and the signal at 4.41 ppm to methylene protons H7 bond to the acid group (Fig. 2a), in accordance with Du and Hsieh’s peak attribution [19]. In Fig. 2b-e, a comparison of ^1^H NMR spectra of the reaction product after functionalization of *O*-CMCS with DA changing the weight ratio between *O*-CMCS and DA were reported. New sets of signals, in the aromatic region, 6.55–7.02 ppm, were shown corresponding to the aromatic protons of DA, covalently bonded to the polymer backbone by an amide bond. The signals at 6.55, 6.59, and 6.66 ppm correspond to the three protons of the aromatic ring of DA. The signal sets at 6.75–6.86 ppm and 7.00–7.25 ppm, observed in spectra Fig. 1c and d, and in low percentages in spectrum Fig. 1e, were assigned to the three protons of the DA aromatic ring in two oxidized forms, probably formed during purification operations or product conservation [32].

The percentage of DA incorporated into the polymer, i.e., DS value, increases with the amount of initial neurotransmitter DA used (Table 2). For instance, starting with 50 mg of *O*-CMCS and 25 mg of DA, a DS of 11 (± 2) % is obtained (Fig. 2b). This value increases to 14 (± 2) % (Fig. 2c) when using 50 mg of *O*-CMCS and 50 mg of DA. Notably, as expected, higher DS values are achieved at specific *O*-CMCS to DA mass ratios: a 1:2 ratio (50 mg and 100 mg, respectively) yields a DS of 24 (± 2) % (Fig. 2d), while a 1:3 ratio (33 mg and 100 mg, respectively) results in a DS of 27 (± 2) % (Fig. 2e).

The DS % values of *O*-CMCS-*g*-DA conjugates **1a-d** were also determined by chemical hydrolysis under strong acidic conditions and the obtained results are also reported in Table 2. As can be seen, the DS % values of *O*-CMCS-*g*-DA conjugates **1a-d** were higher than those observed by ^1^H NMR spectroscopy, even though the rank order of the DS% values for conjugates **1a-d** was the same employing both the approaches. Hence, the DS% values for conjugates **1a-d** measured by chemical hydrolysis under strong acidic conditions were overestimated in comparison with those determined by ^1^H NMR spectroscopy whereas, under the same experimental conditions, the *O*-CMCS-*g*-DA conjugate **1e** (Table 2), previously described by us, resulted underestimated enough [17]. Overall, the present results support the suggestion already made that, most probably, the conditions used for hydrolysis under strong acidic environment should be revised to obtain appropriate hydrolysis of the starting conjugates. On the other hand, to determine the DS% value of conjugate **1c**, we employed a method based on potentiometric titration after alkaline hydrolysis according to a procedure previously described [20, 21]. In that case, we determined a DS % value of 20.3 (± 0.3), which is, globally, close to the value measured by ^1^H NMR spectroscopy (i.e., 27 ± 2). It may be indicative that DS% determination under the conditions described for the basic hydrolysis should be reliable.

### Thermogravimetric analysis (TGA)

**Fig. 3:**
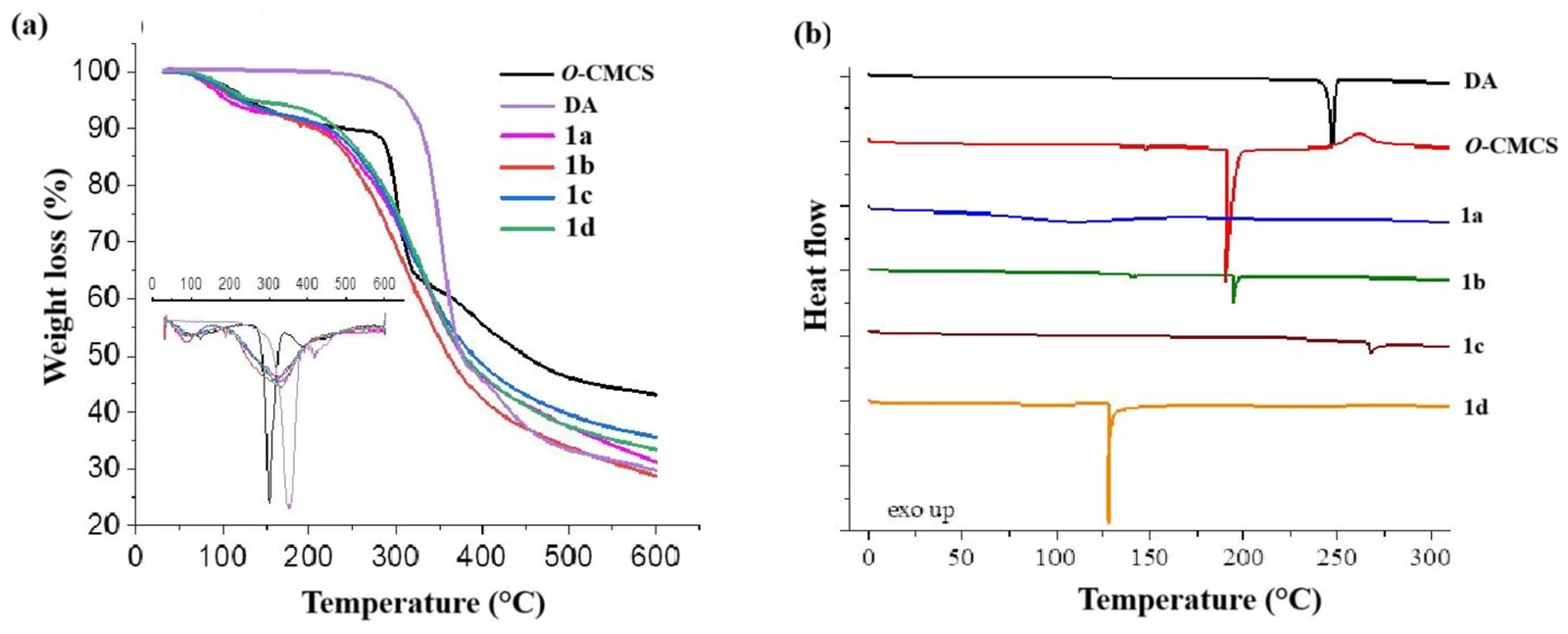
Panel (**a**): TGA traces of *O*-CMCS, DA; *O*-CMCS-*g*-DA **1a**, *O*-CMCS-*g*-DA **1b**, *O*-CMCS-*g*-DA **1c**, *O*-CMCS-*g*-DA **1d**; panel (**b**): DSC thermograms of DA, *O*-CMCS, *O*-CMCS-*g*-DA **1a**, *O*-CMCS-*g*-DA **1b**, *O*-CMCS-*g*-DA **1c**, *O*-CMCS-*g*-DA **1d**

Figure 3a reports the thermogravimetric plots of the synthesized materials and all the main evens observed in studies are summarized in Table 3. While DA showed a single thermal event of decomposition centered at 353 °C, all other samples exhibited a weight loss ascribable to the water presence (8–10%) typical of the polysaccharides. Pure *O*-CMCS polymer presented two main decomposition events: the first one (sharp) at 295 °C attributed to the decarboxylation of the functionalized chitosan unit (corresponding to ~ 30% weight loss) and the second one (broad) at 390 °C associated with the saccharide scaffold decomposition. Conversely, along with the volatile/water removal, the four tested *O*-CMCS-*g*-DA conjugates only exhibited broad decomposition steps centered at 303–330 °C, so confirming the amide functionalization of the carboxylic group belonging to *O*-CMCS.

**Table 3:** Water/volatiles content, main temperature of decomposition (Td) with the relevant weight losses percentages and residues at 600 ◦C of DA, *O-*CMCS as well as the relevant of conjugates **1a-d**

Sample	Water/Volatiles (%)	Td (^◦^C)/weight loss (%)	Residue at 600 ^◦^C (%)
DA	-	353/51	29.8
*O*-CMCS	10.8	295/37.0; 387.7/20.2	42.8
**1a**	6.1	330/68.7	31.3
**1b**	9.1	303.5/71.4	28.6
**1c**	9.4	316.7/64.5	35.5
**1d**	4.7	329.1/66.7	33.3
**1e** ^a^	13 ^a^	304.9/56.3 ^a^	30.5 ^a^

^a^ From Ref [17]

### Differential scanning calorimetry (DSC)

DSC curves of different polymers/conjugates are shown in Fig. 3b. DA and *O*-CMCS exhibited a melting point at 247 °C and 190 °C, respectively, whereas in the case of the conjugate **1a** the disappearance of both melting events was noticed, suggesting the existence of an amorphous solid state. Interestingly, the DSC thermograms of the other conjugates were different from the one of the conjugate **1a**. In the case of the DSC curve of the conjugates **1b** and **1d** semicristallinity was detected from the disappearance of the melting point of the neurotransmitter, but the shift at lower temperature of the *O*-CMCS melting point occurred (at 194 °C and 128 °C for the conjugates **1b** and **1d**, respectively, (Fig. 3b). Moreover, for the conjugate **1c** the semicrystalline solid state was also deduced from DSC profile, although the melting point of DA was shifted in the DSC thermogram of the conjugate **1c**, being shown at 268 °C together with the disappearance of the *O*-CMCS melting point (Fig. 3b).

### SEM microscopy

**Fig. 4:**
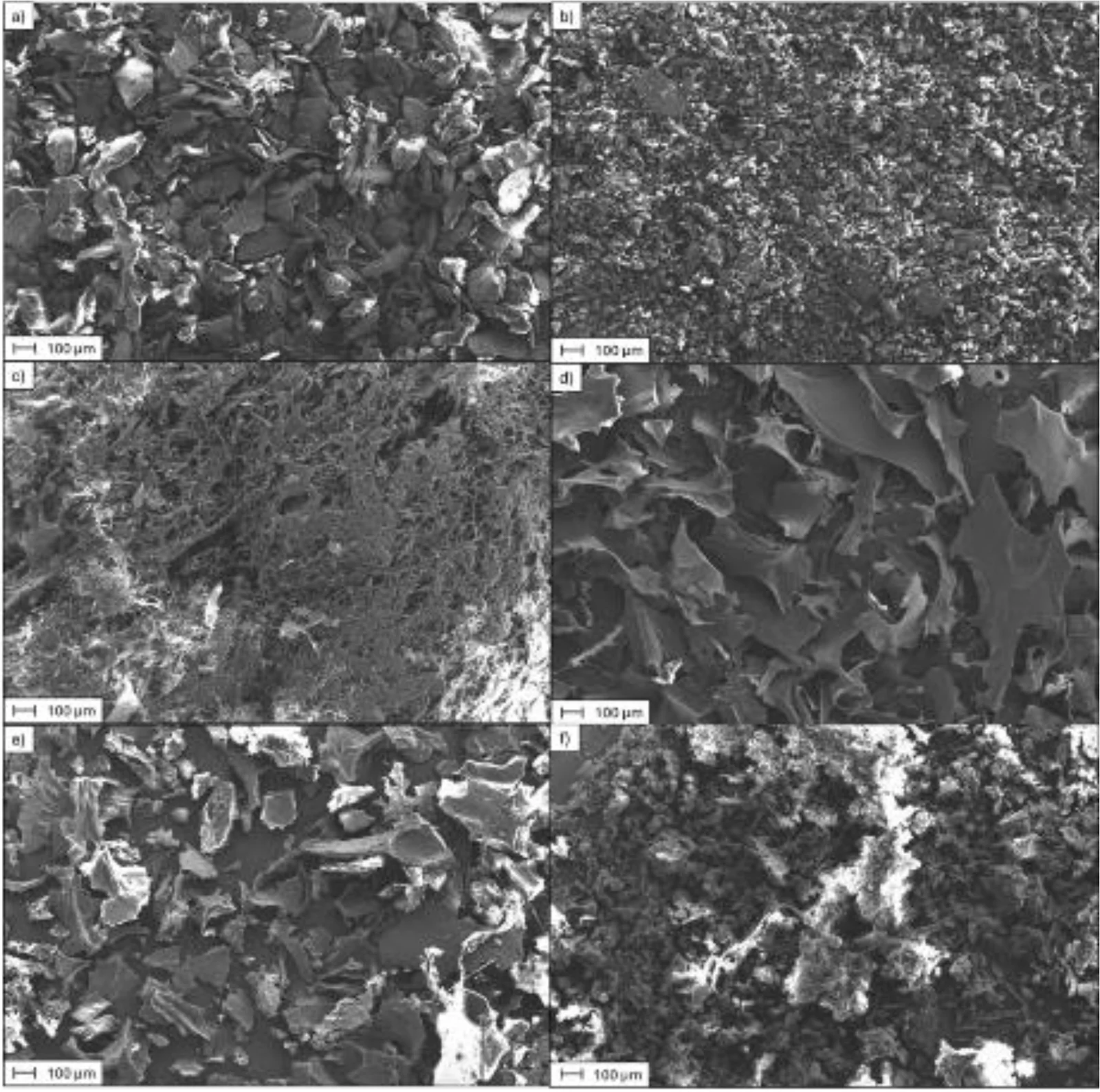
Scanning electron microscopy (SEM) images at 100x magnification of **a**) pure *O*-CMCS, **b**) pure DA, **c**) conjugate **1d**, **d**) conjugate **1a**, **e**) conjugate **1b** and **f**) conjugate **1c**

The morphology of the samples was observed by SEM and reported in Fig. 4. Images for pure components were acquired for comparison and revealed a flake-like morphology for pure *O*-CMCS (Fig. 4a) and microcrystalline structures for pure DA (Fig. 4b), which were not observed in the conjugated samples. The conjugate **1d** (Fig. 4c) exhibited a fibrous and continuous structure [33] while **1a** showed the presence of lamellae with smooth surfaces, covering sample areas of several hundred µm^2^ and thickness below 1 μm (Fig. 4d). This morphology is preserved in **1b**, however, the lamellae show reduced structure area and an increased thickness of few µm (Fig. 4e). Another interesting observation is related to the presence of smaller aggregates that increases the irregularity of the surface if compared to **1a**. In **1c** (Fig. 4f) the lamellar structure fragmented into smaller aggregates with less defined morphology compared to the previous samples and showed highly irregular surfaces.

Energy-dispersive X-ray (EDX) analyses were all conducted on all conjugated samples as well as on pure *O*-CMCS and DA as reference materials. While a quantitative analysis was not pursued on carbon, oxygen and nitrogen due to the inherent limitations of EDX in accurately detecting light elements [34], it could be noticed that the concentration of Na present in pure *O*-CMCS (18 ± 1 Wt%) and Cl^−^ present in pure DA (18.7 ± 1 Wt%), as counter ions of carboxylate and DA, respectively, were only present in traces in all **1a-d** conjugates due to the amide functionalization of the carboxylic group of *O*-CMCS polymer (Fig. S1 and Table S1, Supplementary Information). Moreover, it is noteworthy that the conjugates **1a-d** showed average weight percentages of nitrogen ranging from 3.61 to 5.94 Wt%, being the differences among them, however, not statistically significant As expected, due to the aforementioned limitations of EDX in detecting light elements, these data do not support quantitative evaluation or correlation with the DS value (Table S1 Supplementary Information).

### In vitro drug release from conjugates 1a-d

**Fig. 5:**
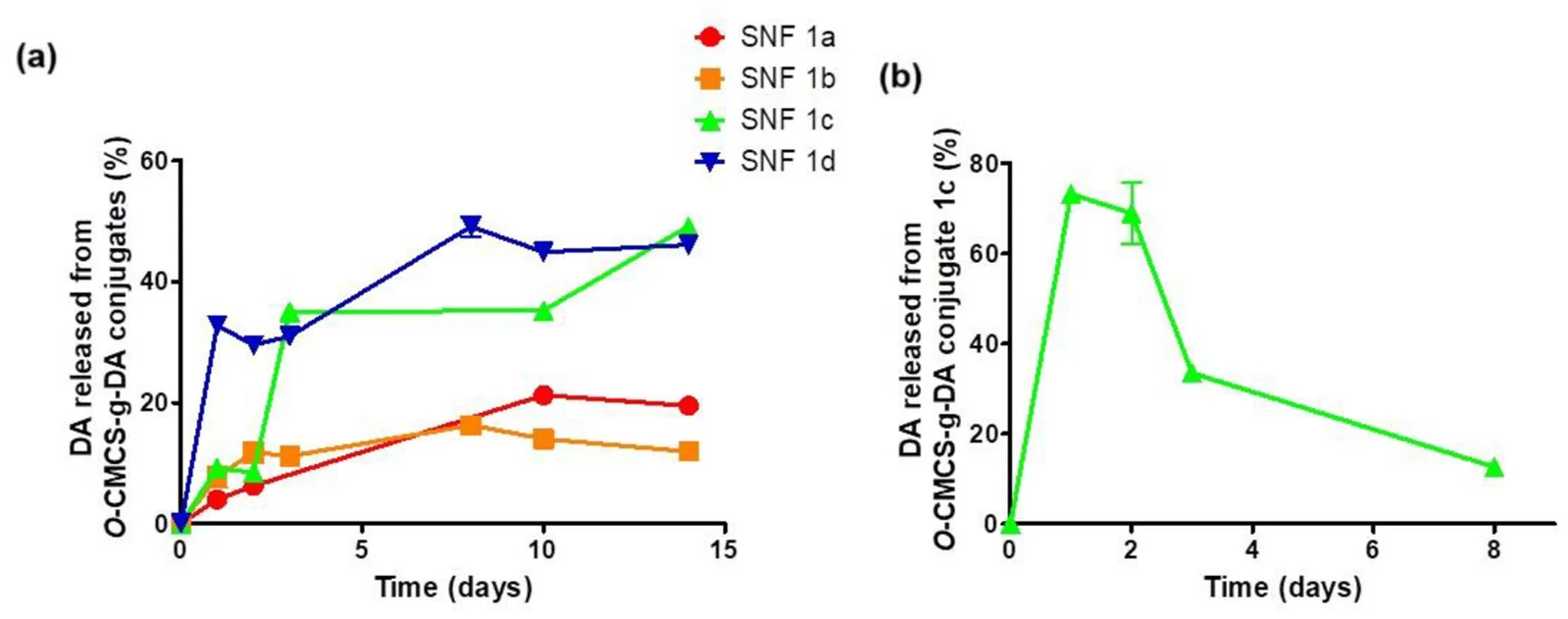
Panel (**a**): DA released in SNF (pH 6) at 37 ± 0.1 °C from conjugates **1a-d**; Panel (**b**): DA released from conjugate **1c** in PBS at brain pH of 7.2 [35] and at 37 ± 0.1 °C

All investigated conjugates **1a-d** were evaluated for the neurotransmitter delivered in in vitro tests in SNF (pH 6.0 without enzymes) as release medium at 37 ± 0.1 °C and the observed results are shown in Fig. 5a from which a sustained release profile was detected in any case. Moreover, from the conjugate prepared at the lowest w/w ratio polymer: DA, i.e., conjugate **1c** as well as the conjugate **1d**, namely that prepared in excess of polymer, the neurotransmitter amount released after 14 days was more than the double compared to that from **1a** and **1b**. More precisely, the conjugate **1c** showed the highest percentage of the neurotransmitter delivered (i.e., after 14 days about 46% of DA was released), whereas the conjugate **1b** provided the lowest amount of DA delivered, namely 12% at the latest time point (Fig. 5a). Remarkably, through the study, no change of colour of SNF medium was ever detected for all the conjugates examined. However, as already observed in the case of the conjugate **1e** (Table 2) previously investigated [17], also all conjugates herein studied exhibited degradation products of the neurotransmitter as additional HPLC peaks only when HPLC chromatograms were acquired from 10 to 14 days with retention times over 6 min. Overall, such degradation peaks were negligible, being SNF buffered at pH 6 and it is well known that, in acidic medium, the DA autoxidation is successfully controlled [31]. Furthermore, in the case of the conjugate **1c**, the in vitro release test was also carried out at brain pH of 7.2 to assess the pH sensitivity of this nanocarrier (Fig. 5b) [35]. As shown in Fig. 5b, the kinetic release profile reached the maximum DA amount delivered (i.e., 68%) after 2 days of release time and, then, a collapse of the profile was detected. The study was stopped after 1 week of incubation of the conjugate **1c** in PBS (pH 7.2) because the buffer started to change colour to grey-black, clearly suggesting DA autoxidation [31].

### Self-aggregation of *O*-CMCS-g-DA conjugate 1c to give polymeric micelles and determination of their critical micelle concentration (CMC)

**Fig. 6:**
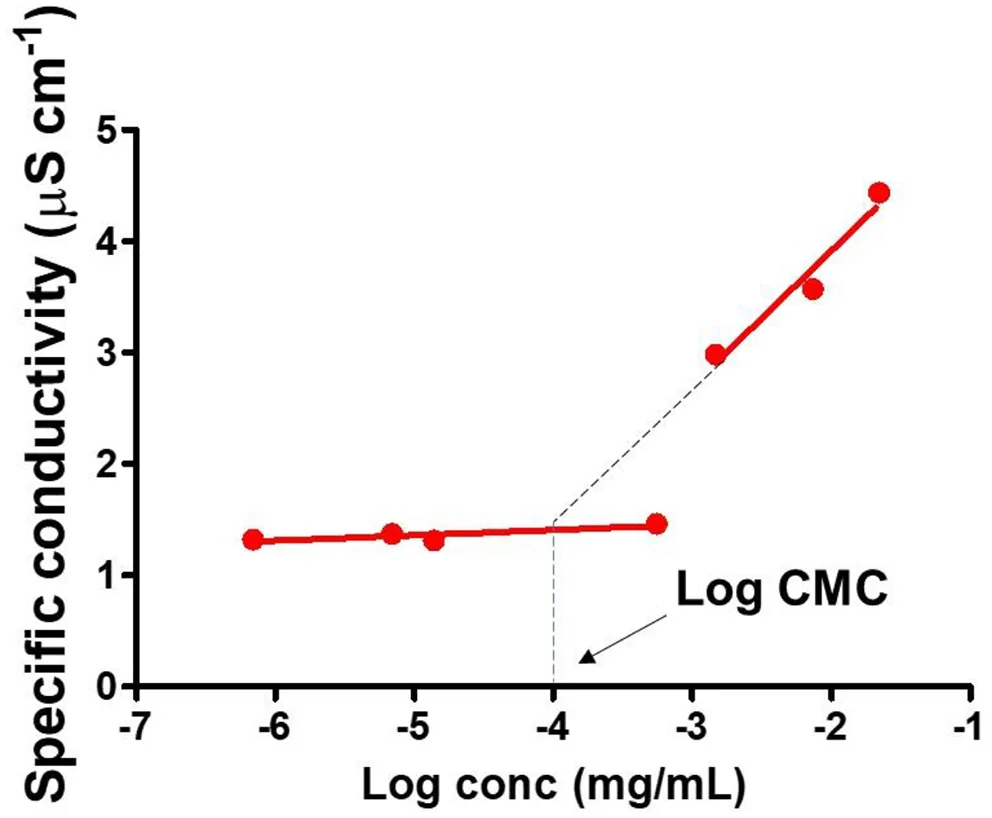
Specific conductivity plotted against logarithm of *O*-CMCS-*g*-DA conjugate **1c** concentration

Given the amphiphilic nature of the conjugates of *O*-CMCS-*g*-DA, due to the hydrophobic contribute of the aromatic rings of the catechol moieties and the hydrophilic characteristic of the polysaccharide backbone, we aimed at assessing if the conjugate at highest DS (i.e., **1c**) can spontaneously form micelles in aqueous environment. For this purpose, we employed the dialysis method as detailed in Materials and Methods section. The CMC determination of the resulting polymeric micelles obtained starting from conjugate **1c** was performed employing the conductometric method based on the sudden change of specific conductivity when micelle formation occurs. This simple and easily reproducible method provided the CMC value by graphical way evaluating the abscissa corresponding to the intersection between straight lines of specific conductivity against the logarithmic concentration of the copolymer (Fig. 6). In this way, we measured a CMC value for *O*-CMCS-*g*-DA micelles prepared starting from the conjugate **1c** of 1 × 10^− 4^ mg/mL.

QUE, a naturally occurring polyphenolic flavonoid endowed with efficient antioxidant activity and selected by us as a lipophilic (calculated log P 1.5, from PubChem) antioxidant model drug, was encapsulated into *O*-CMCS-*g*-DA micelles prepared starting from conjugate **1c** by the dialysis method, and their main physico-chemical properties are summarized in Table 4 together with those of the corresponding unloaded micelles. The obtained micelles were seen to be endowed with an average particle size in the range 299–411 nm and zeta potential values included in the range − 8.3 – -10.7 mV, evidencing that QUE loading increased mean particle size and shielded the negative charges of the unconjugated -COO^−^ groups of the CS polymeric backbone (Table 4).

**Table 4:** Physicochemical properties of the micelles prepared by self-assembly of *O*-CMCS-*g*-DA conjugate **1c**^a^

Formulation	Size (nm)	PDI^b^	Zeta Potential (mV)	QUE E.E. (%)
Unloaded micelles	299 ± 23	0.60 ± 0.07	-10.7 ± 0.9	-
QUE-loaded micelles	411 ± 17**	0.50 ± 0.02	-8.3 ± 1.77	18 ± 3

^a^Mean ± SD of at least eight replicates (*n* = 8) are reported. ^b^PDI: polydispersity index. Unloaded micelles were taken as reference for statistical evaluation (**p ≤ 0.001)

### MTT assay

**Fig. 7:**
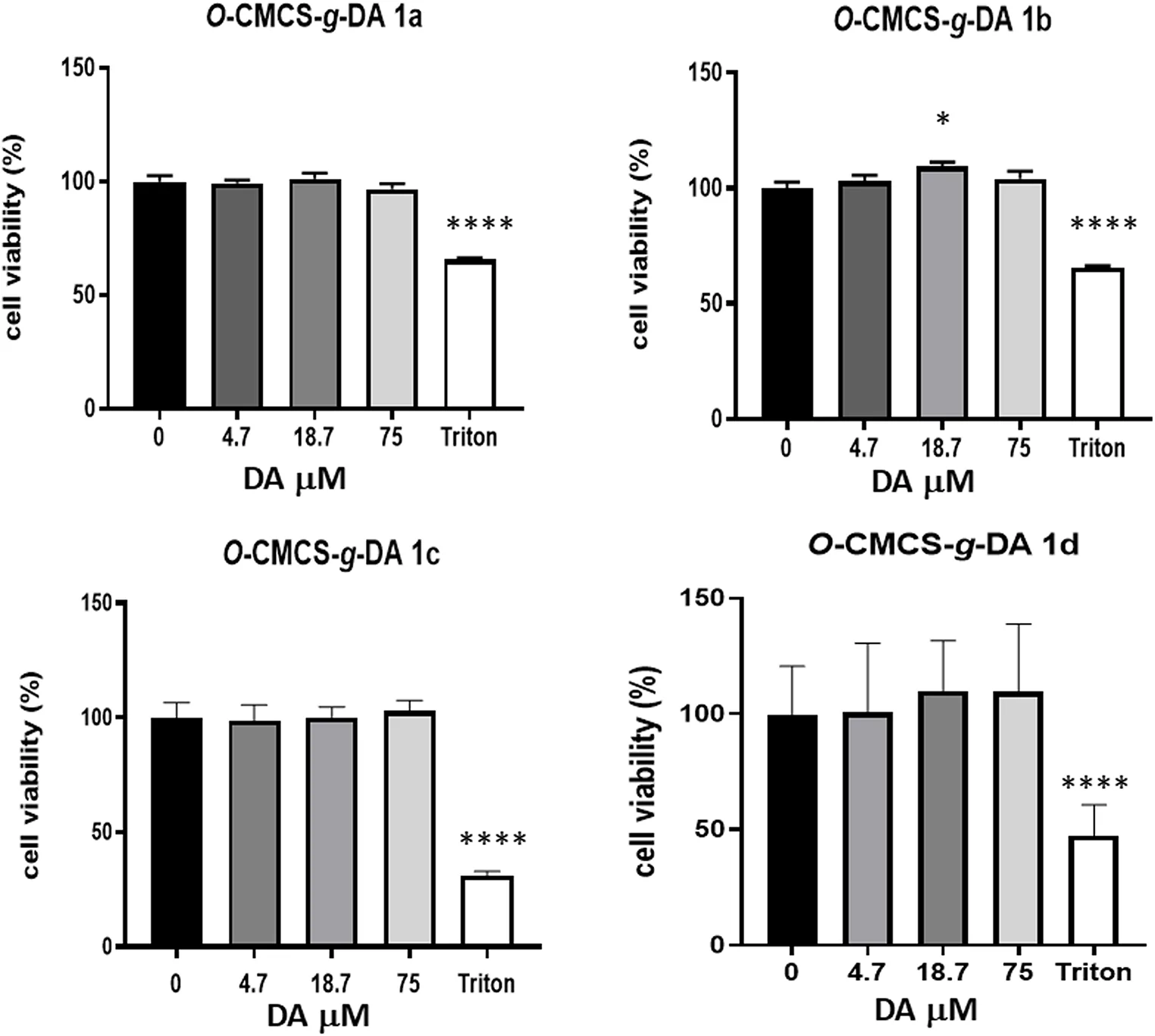
Biocompatibility of *O*-CMCS based formulations. SH-SY5Y were challenged with *O*-CMCS-*g*-DA conjugates at the indicated DA concentrations of 4.7, 18.75, and 75 µM for 24 h. Cells were then assayed for vitality by the MTT assay. Controls are untreated cells (i.e. DA = 100% of vitality), whereas 1% Triton X-100 (Triton) was used as positive control. **** *p* < 0.0001 Triton vs. untreated; *p < 0.05 vs. untreated. Data are the results of two-three experiments each carried out in eight wells

In order to verify the cyto/biocompatibility of conjugates with the target neuronal cells, SH-SY5Y cells were treated with O-CMCS-based conjugates at the indicated concentrations. None of these formulations resulted to decrease cell viability (Fig. 7).

**Fig. 8:**
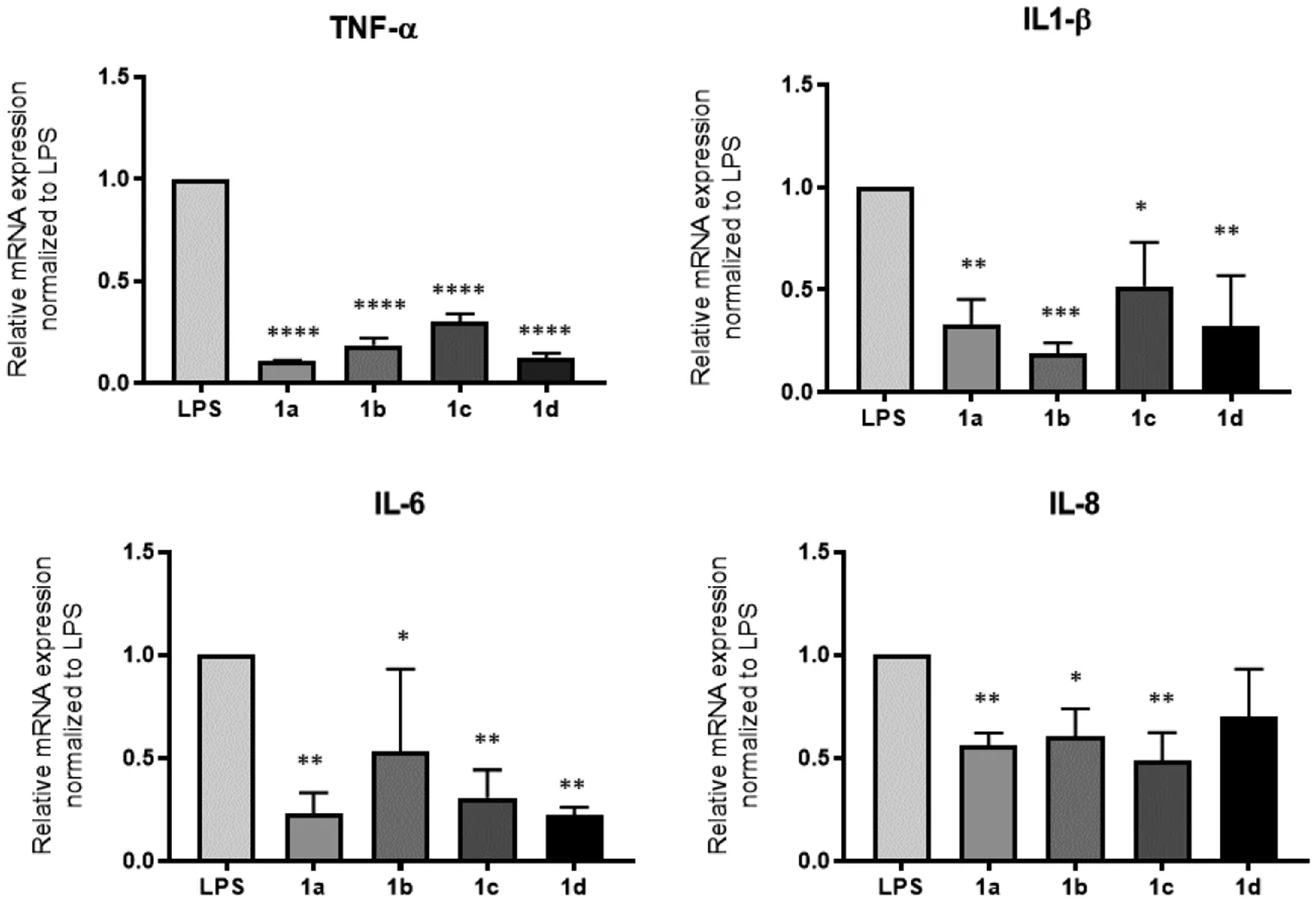
**1a-d** conjugates downregulate cytokine expression in SH-SY5Y cells. SH-SY5Y were treated with both LPS (100 ng/mL) and conjugates for 24 h. Total RNA was extracted and qRT-PCR was performed in order to quantify TNF-α, IL-1β, IL-6, and IL-8 mRNA levels. Cytokine mRNAs were normalized to only-LPS treated cells. Results are shown as fold induction where LPS is equal to 1. Data represent the mean ± SD (*n* = 3). * *p* < 0.05; ** *p* < 0.01; *** *p* < 0.001; **** *p* < 0.0001, all relative to the comparison of LPS samples

### Effects of *O*-CMCS-g-DA 1a-d conjugates on cytokine gene expression

To evaluate the modulation of inflammation involved in PD pathogenesis at the neuronal level, a study of cytokine mRNA levels was carried out in SH-SY5Y cells by incubating them in the presence of both LPS, a molecule generating a robust pro-inflammatory stimulus, and each of *O*-CMCS-*g*-DA conjugates for 24 h. Thus, total RNA was isolated and used for a quantitative Real-Time PCR assay. The results evidenced that, in comparison with the only-LPS treated samples, conjugates abated the expression of TNF-α, IL-1β, IL-6, and IL-8 mRNA levels (Fig. 8). TNF-α was the cytokine which showed the higher sensitivity than the others to the treatment, regardless of the conjugate used.

## Discussion

The main aim of the present work was to prepare and characterize new amphiphilic *O*-CMCS-*g*-DA conjugates able to form stable micelles in aqueous environment and encapsulate in their core lipophilic therapeutic substances acting at different target sites involved in PD, giving rise so to multifunctional nanomedicines to be administered by intranasal route in the brain. For this purpose, we prepared *O*-CMCS-*g*-DA conjugates with increasing DA amounts adopted in the synthetic procedure taking into account that DS is a well-recognized factor influencing the physicochemical features of the resulting polymeric micelles and it was already proved even for other CS-based grafted polymers [36–38]. Furthermore, our choice to pay attention to *O*-CMCS, mainly due to the better performance showed by this polymer in the previous study [17], was also motivated by its biological characteristic as antioxidant suitable for the fabrication of promising drug delivery systems involved in oxidative stress related diseases as PD [39].

Our results showed that the employed synthetic procedure, based on EDC mediated coupling reaction between the amino group of the neurotransmitter DA and the carboxylic function of the *O*-CMCS polymer, works well in aqueous environment. Indeed, all the *O*-CMCS-*g*-DA conjugates **1a-d** were prepared in satisfactory yields ranging from the 17–38%.

The thermogravimetric analytical study was used to evaluate the thermal stability of the *O*-CMCS-*g*-DA conjugates **1a-d** providing information about their mass changes on increasing the temperature. In general, our findings align with the thermal behaviour reported for polysaccharide-based materials, particularly chitosan derivatives under thermal stress [40].

The results obtained for these four conjugates are consistent with that suggested for the previously examined one (i.e., **1e**) [17]. Indeed, all the four conjugates **1a-d** showed thermal stability up to about 190 °C and the main structural changes due to temperature increase of **1a-d** could be deacetylation and decomposition of the parent polymer chitosan. In addition, it may be hypothesized that the cleavage of substituent groups in the *O*-CMCS-*g*-DA conjugates **1a-d** occurs within the temperature range of 270–330 °C. Besides this, the thermal analysis by DSC showed that the solid state of conjugates **1a-d** ranges from a fully amorphous as for conjugate **1a** to a semicrystalline state as for **1b-d.**

An interesting result of the present study concerns the in vitro neurotransmitter release from conjugates **1a-d** carried out in SNF (pH 6.0 without enzymes) selected as biomimetic release medium at 37 °C and, hence, for its better predictive capability about the in vivo behaviour of these conjugates. In addition, although the accepted mean residence time of a formulation in the nasal cavity is in the range 1–4 h [41], the in vitro release study in SNF herein carried out for a longer time (up to 14 days) to check the possible formation of neurotransmitter degradation products. More precisely, it was found that conjugates **1c** and **1d**, namely those prepared in excess of DA and polymer, respectively, the neurotransmitter amount released after 14 days was more than the double compared to that delivered from each of **1a** and **1b** conjugates without any trace of DA degradation products. Interestingly, it may be considered an advantageous aspect in the perspective of using such materials for DA brain delivery. At present, it is a difficult and intriguing task to find the rationale behind the marked increase of DA release from conjugates **1c**,**d** in comparison with that occurring from **1a**,** b**, since several factors, including lamellar/fibrous morphology and amorphism/crystallinity character, are to be considered. Our hypothesis, to be fully demonstrated, is that such result can be rationalized taking into account, mainly, of the DS value and the polymer/DA (w/w) ratio because both of them play a crucial role to determine the amphiphilic character of these conjugates. Thus, the higher DA release from **1c** can be explained considering that it is characterized by the highest DS value and hence the highest content in neurotransmitter, while conjugate **1d**, being endowed with the highest polymer/DA (w/w) ratio, is hydrophilic enough to be quite soluble in the release medium. However, this hypothesis needs further experimental information including, for instance, that on the swelling properties of all the conjugates **1a-d** to be fully proved. These studies led us to conclude that conjugate **1c** is appropriate for brain delivery through the intranasal route of administration since it allows a sustained release of about 46% of neurotransmitter after 14 days.

Besides that, it is also noteworthy that DA release from **1c** increased up to 68% at brain pH of 7.2 (against the nasal pH of 6.0) after 2 days of release time confirming so the pH-sensitive nature of this conjugate **1c**. This can be accounted for considering that protonation of carboxylate groups of *O*-CMCS in acidic conditions hinders the swelling of the polymeric chains of *O*-CMCS, whereas the ionization of carboxylic acid function at pH 7.2 facilitates the swelling of the same polymer increasing the DA release rate. However, as shown in Fig. 5b, a fast decrease in DA release was noted after one day of release at brain pH of 7.2 very probably due to the quick degradation of the neurotransmitter in neutral-alkaline medium [31].

A further remarkable outcome of this paper concerns the evidence gained that *O*-CMCS-*g*-DA conjugate **1c** can spontaneously form micelles in aqueous medium due to the hydrophobic and hydrophilic character of the catechol moiety and CS backbone, respectively. As already mentioned in Introduction section, we were unable to demonstrate such self-assembled system for the *O*-CMCS-*g*-DA conjugate previously prepared (i.e., **1e**, Table 2) [17] and it was probably due to the low substitution degree (DS) of the examined conjugate **1e**. Therefore, we focused our attention to the conjugate **1c**, namely that endowed with the highest DS value for which the CMC value of 1 × 10^− 4^ mg/mL was determined by the intersection between the straight lines of the plot of the specific conductivity against the logarithmic concentration of the conjugate **1c**. It should be noted that this CMC value is slightly lower than those measured for other CS-based polymeric micelles [26, 28, 36, 42] suggesting a good stability of these micelles upon dilution by body fluids and blood circulation. The CMC value, indeed, is a very important feature of polymeric micelles because it is indicative of their stability in body fluids and in blood circulation where they undergo a large dilution and, therefore, may change their morphology and drug encapsulation efficiency. From Table 4 it can be deduced that unloaded micelles presented an average diameter of around 299 nm, while when QUE was encapsulated, the size increased to around 411 nm. Moreover, in any case high PDI values were observed, suggesting that a certain variability in micelle size occurred. Interestingly, a similar micellar size enhancement and high PDI values compared to the corresponding unloaded counterpart were already described in literature with other QUE-loaded *O*-carboxymethyl chitosan-cholic acid conjugates (*O*-CMCSCA) -based self-aggregates in aqueous environment by Du et al. [43]. These authors pointed out that both the effects are depending on the starting drug/carrier weight ratio employed and this ratio play a key role in determining suitable characteristics of these nanocarriers. As for zeta potential values, from Table 4 it can be deduced that both QUE-loaded and unloaded micelles are endowed with slightly negative surface charge (i.e., -8.3 mV and − 10.7 mV at pH 6, respectively). These values are not significantly different among themselves from a statistical point of view, suggesting that QUE loading does not affect the surface charge as may be expected with an encapsulation of the antioxidant agent QUE within the core of the micelles (Fig. S2, Supplementary information). Moreover, these values are very close to -13 mV observed at pH 6.2 for the QUE-loaded *O*-CMCSCA-based self-aggregates evaluated by Du et al. [43]. As for the encapsulation efficiency of QUE (QUE E.E.%), it is not very high being of 18 ± 3% as can be seen from Table 4. However, it is well known that also this parameter is strictly depending on the starting drug/polymer weight ratio employed [44] and, in future studies, we planned to improve QUE E.E.% values varying the mentioned ratio. Therefore, it can be concluded that such *O*-CMCS-*g*-DA based polymeric micelles may be considered as promising multifunctional nanomedicines to be administered intranasally for brain delivery of the neurotransmitter DA together with an antioxidant substance as QUE involved in the oxidative stress. In order to assess a translational-oriented approach to PD by *O*-CMCS-*g*-DA conjugates, we evaluated cyto/biocompatibility and inflammatory responses in neurons. Not only conjugates **1a**-**d** were not cytotoxic, but also dampened neuronal transcription of inflammatory cytokines genes when SH-SY5Y cells were stimulated with a pathogen-associated molecular pattern (PAMP), i.e. LPS, a well-known PD-mimetic [45]. SH-SY5Y cells can respond to PAMPs, including LPS, significantly at 24-h [46], as in our study, however, to mimic chronic inflammation, a long-term exposure to LPS should be evaluated.

## Conclusions

In this paper, some *O*-CMCS-*g*-DA conjugates **1a-d** with different DS values were prepared and structurally characterized by FT-IR, ^1^H-NMR and hydrolytic reactions. The *O*-CMCS-*g*-DA conjugate at highest DS value **1c** was proved to self-assembly forming polymeric micelles with a CMC value of 1 × 10^− 4^ mg/mL and an average diameter of 299 ± 23 nm. Moreover, it was shown that such micelles were able to encapsulate the lipophilic polyphenolic flavonoid antioxidant QUE giving rise to polymeric micelles with a surface charge not statistically different from that observed with the unloaded counterpart, according to a drug encapsulation within the micellar core. None of the conjugates **1a-d** was cytotoxic against the target neuronal SH-SY5Y cells, at the indicated concentrations Moreover, all the *O*-CMCS-*g*-DA conjugates **1a-d** were able to downregulate neuroinflammation as demonstrated by genetic analysis tools. Therefore, it can be concluded that such *O*-CMCS-*g*-DA based polymeric micelles arising from the conjugate **1c** may be considered promising multifunctional nanomedicines to be administered intranasally to the brain parenchyma for the co-delivery of the neurotransmitter DA and an antioxidant substance involved in the oxidative stress as QUE. Importantly, it occurs exploiting a single nanocarrier endowed with modulating properties towards neuroinflammation. Such findings disclose new advantageous perspectives and options to reduce oxidative stress and neuroinflammation for diagnosis and therapeutic treatment of PD. Moreover, they warrant further studies for evaluating the in vivo efficacy of these conjugates with properly selected animal models and the relative results will be presented in due course.

## Supplementary Information

Below is the link to the electronic supplementary material.

## Data Availability

All data generated or analysed during this study are included in this published article.
