# Supplementary material for: Synthesis and characterization of chitosan-grafted-dopamine based micelles as multifunctional nanomedicines for Parkinson’s disease treatment by intranasal administration
Source: Drug Deliv Transl Res. 2025 Sep 1;16(9):3310–26. doi: 10.1007/s13346-025-01963-0 (PMC13538208; doi:10.1007/s13346-025-01963-0)
Supplement: Supplementary file 1 [file 13346_2025_1963_MOESM1_ESM.docx]

**Supplementary Information of**

**Synthesis and characterization of chitosan-grafted-dopamine based micelles as multifunctional nanomedicines for Parkinson’s disease treatment by intranasal administration**

**Adriana Trapani^1^* Annalucia Carbone^2^** **Sante Di Gioia^2^ Giuseppe Fracchiolla^1^ Piera Soccio^3^ Filippo Maria Perna^1^, Andrea Francesca Quivelli^1^ Gian Paolo Suranna^4^ Roberto Grisorio^4^ Chiara Lo Porto^4^ Daniele Conelli^4^ Giuditta Colangelo^1^ Massimo Conese^2^**

^1^Department of Pharmacy-Drug Sciences, University of Bari “Aldo Moro”, via E. Orabona, 4-70125 Bari, Italy

^2^Department of Clinical and Experimental Medicine, University of Foggia, 71122, Foggia, Italy

^3^Department of Medical and Surgical Sciences, University of Foggia, 71122, Foggia, Italy

^4^Department of Civil, Environmental, Land, Building Engineering and Chemistry (DICATECh), Polytechnic University of Bari, via E. Orabona 4, I-70125 Bari, Italy

^*^Corresponding Author: Prof. Adriana Trapani

Department of Pharmacy-Drug Sciences

University of Bari “Aldo Moro”, Bari, Italy

Tel.: +39-0805442114. Fax: +39-0805442724.

Email: [adriana.trapani@uniba.it](mailto:adriana.trapani@uniba.it)

**Table S1.** Quantitative elemental analysis results showing weight percentages (Wt%) and their associated standard deviations (SD) as determined by EDX for pure DA, pure *O*-CMCS, and conjugates**1a-d.**

| **Sample** | **C**  **(Wt% ± SD)** | **N**  **(Wt% ± SD)** | **O**  **(Wt% ± SD)** | **Na**  **(Wt% ± SD** | **Cl**  **(Wt% ± SD)** |
| --- | --- | --- | --- | --- | --- |
| DA | 51.87 ± 1.29 | 1.51 ± 1.48 | 27.94 ± 1.07 | 0.00 ± 0.00 | 18.68 ± 0.53 |
| *O*-CMCS | 29.41 ± 2.54 | 0.00 ± 0.00 | 46.05 ± 2.53 | 18.16 ± 1.48 | 6.37 ± 0.69 |
| **1a** | 55.94 ± 1.39 | 3.61 ± 2.02 | 40.18 ± 1.67 | 0.20 ± 0.18 | 0.07 ± 0.07 |
| **1b** | 41.04 ± 0.70 | 5.94 ± 1.12 | 52.77 ± 0.83 | 0.17 ± 0.83 | 0.08 ± 0.04 |
| **1c** | 40.40 ± 0.83 | 4.68 ± 1.34 | 54.37 ± 0.99 | 0.28 ± 0.16 | 0.27 ± 0.06 |
| **1d** | 34.46 ± 0.91 | 5.52 ± 1.46 | 58.35 ± 1.20 | 1.60 ± 0.27 | 0.07 ± 0.08 |

**Fig. S1** Results obtained from scanning electron microscopy (SEM) coupled with an energy dispersive X-ray spectroscopy (SEM-EDX) for DA, *O*-CMCS, and conjugates **1a-d.**

**Fig. S2** Schematic representation of a QUE-loaded *O*-CMCS-*g*-DA polymeric micelles
